# Online Reviews as Health Data: Examining the Association Between Availability of Health Care Services and Patient Star Ratings Exemplified by the Yelp Academic Dataset

**DOI:** 10.2196/publichealth.7001

**Published:** 2017-07-12

**Authors:** Nam N Tran, Joon Lee

**Affiliations:** ^1^ Health Data Science Lab School of Public Health and Health Systems University of Waterloo Waterloo, ON Canada

**Keywords:** Yelp, health care access, health care availability, patient satisfaction, patient rating, patient experience, open hour, clinic hour, online reviews

## Abstract

**Background:**

There have been public health interventions that aim to reduce barriers to health care access by extending opening hours of health care facilities. However, the impact of opening hours from the patient’s perspective is not well understood.

**Objective:**

This study aims to investigate the relationship between temporal accessibility of health care services and how patients rate the providers on Yelp, an online review website that is popular in the United States. Using crowdsourced open Internet data, such as Yelp, can help circumvent the traditional survey method.

**Methods:**

From Yelp’s limited academic dataset, this study examined the pattern of visits to health care providers and performed a secondary analysis to examine the association between patient rating (measured by Yelp’s rating) and temporal accessibility of health care services (measured by opening hours) using ordinal logistic regression models. Other covariates included were whether an appointment was required, the type of health care service, the region of the health care service provider, the number of reviews the health care service provider received in the past, the number of nearby competitors, the mean rating of competitors, and the standard deviation of competitors’ ratings.

**Results:**

From the 2085 health care service providers identified, opening hours during certain periods, the type of health care service, and the variability of competitors’ ratings showed an association with patient rating. Most of the visits to health care service providers took place between normal working hours (9 AM-5 PM) from Sunday to Thursday, and the least on Saturday. A model fitted to the entire sample showed that increasing hours during normal working hours on Monday (OR 0.926, 95% CI 0.880-0.973, *P*=0.03), Saturday (OR 0.897, 95% CI 0.860-0.935, *P*<0.001), Sunday (OR 0.904, 95% CI 0.841-0.970, *P*=0.005), and outside normal working hours on Friday (OR 0.872, 95% CI 0.760-0.998, *P*=0.048) was associated with receiving lower ratings. But increasing hours during outside normal working hours on Sunday was associated with receiving higher ratings (OR 1.400, 95% CI 1.036-1.924, *P*=0.03). There were also observed differences in patient ratings among the health care services types, but not geographically or by appointment requirement.

**Conclusions:**

This study shows that public health interventions, especially those involving opening hours, could use crowdsourced open Internet data to enhance the evidence base for decision making and evaluation in the future. This study illustrates one example of how Yelp data could be used to understand patient experiences with health care services, making a case for future research for exploring online reviews as a health dataset.

## Introduction

There have been attempts to reduce the physical barrier to health care access by offering extended opening hours, such as the incentive schemes introduced by the UK National Health Service (NHS) to extend hours of general practices [1-3]. Similar strategies are also found in countries like the Netherlands and New Zealand, but they are not as common in the United States [4-6]. Previous studies have found that increasing opening hours may lead to an increase in patient satisfaction or rating in surveys, and the distribution of opening hours may be more important than total opening hours per week [1,7]. A previous study examining extended hours of general practice in the United Kingdom suggested that there might be a possible association between the inability to take time away from work and lower patient experience [2]. Other emerging evidence suggested that increasing opening hours may have other potential benefits, such as reducing the demand of emergency department visits [3,8-10], avoiding delays in patients seeking care [11], and encouraging people to seek preventive health checks [12], all of which could potentially contribute to an increase in patient satisfaction or survey rating.

One of the challenges in conducting similar research about the temporal barriers to health care access is that questionnaires or interviews are the traditional methods of collecting data [1-3,7-9,12,13]. With the rise of online review websites, similar research questions may be answered using open Internet data such as those from Yelp, an online review website that is popular in the United States. This approach, if effective, may offer a novel strategy to assess patient-centered quality of care, while helping to reduce the burden and cost of surveying.

There are concerns that reviews on commercial websites, such as Yelp, are biased and may not reflect the quality of care delivered because the reviewers lack medical expertise [14,15]. However, it has been observed that consumer reviews on commercial websites may offer meaningful evaluations of quality of hospital care [14,16,17]. A previous study has found that hospitals’ Yelp ratings show a high correlation with the Hospital Consumer Assessment of Healthcare Providers and Systems (HCAHPS) survey, the industry standard for assessing hospital patients’ experiences in the United States [14]. Similar to the HCAPHS, higher Yelp scores have shown correlations with lower mortality rates for myocardial infarction, pneumonia, and lower readmission rates for multiple other conditions [14]. In another study, Yelp data were shown to provide additional information to complement the HCAHPS survey because some topics with a strong correlation with Yelp ratings are not measured or reported by HCAHPS [17]. Another study examining Yelp reviews for emergency departments found that they contain similar themes to surveys of inpatient and specific to emergency care settings, thus may offer a new strategy to measure quality from patients’ points of view [18]. Other examples of using online reviews as data about the patient experience include WebMD in studying the quality of physicians from the patient’s perspective [19], Yelp and RateMDs in studying long-term relationships between patients and physicians [20], and HealthGrades, Vitals, and RateMDs in studying factors associated with high ratings of hand surgeons [21].

In this study, we aim to explore whether opening hours are associated with patient ratings of health care service providers. This study chose Yelp over other online review websites because Yelp offers a free limited dataset for research purposes and Yelp is known to have deployed an industrial-scale fake review filter since 2005 [22,23]. Examinations of Yelp’s filter by previous studies suggest that Yelp’s filter is not perfect, but is reasonably effective at detecting fake or fraudulent reviews [23,24].

## Methods

Research ethics approval was not required for this study because the data were from the public domain and offered for free from Yelp.

### Yelp Academic Dataset

Yelp users can submit reviews for businesses listed on Yelp by providing a numerical rating ranging from 1 to 5 stars, similar to a Likert scale in a survey, and a free-text comment. The primary outcome of interest for this study was the overall rating of health care service providers, reported by Yelp as the mean of all nonfiltered individual ratings. This study assumed that reviews submitted for health care service providers were made by patients who had visited the provider at least once and that fake reviews were removed by Yelp’s filter.

Yelp also allows users to voluntarily check-in at a business using mobile devices in exchange for occasional discounts or some other loyalty rewards. In essence, check-in data provide a record of patient visits to health care service providers, which also makes Yelp data uniquely different than traditional survey data. Yelp provided the check-in data as a summary of the total number of check-ins at each business for each 1-hour window throughout the day. Because these data are the total count of check-ins during an undefined period of time, they are not suitable to be used to determine the busy or slow periods for a defined period of time, such as mean weekly or daily. However, the total number of check-ins can still give a rough overview of the busy and slow periods for health care service providers in the sample.

The limited dataset for this study was provided by Yelp for research purposes and a student competition organized by the company [22]. This study obtained version 8 of the dataset in September 2016 [22]. Yelp’s raw data was processed and analyzed using the jsonlite package in R Studio version 1.0.136 [25]. This study analyzed health care service businesses from the dataset that contained 2,685,066 reviews submitted for 85,901 businesses, and 98 attributes for each listed business (eg, address, location, hours, amenities, parking availability) [22]. This limited dataset provided businesses from selected cities in the United Kingdom, Canada, Germany, and the United States. However, this study only considered those from the United States to reduce the effect of the differences in health care systems.

### Sampling for Health Care Service Providers From the Yelp Limited Dataset

Yelp labels each business in a category (eg, restaurant, coffee and tea, family practice). A previous study identified 26 categories on Yelp that are health care services [26]. However, many of the labels overlap with one another (eg, “eyewear and opticians,” “optometrists,” and “laser eye surgery/Lasik”), and most businesses are labeled with more than one. Therefore, based on initial observations of the dataset, this study created a search strategy to identify, verify, and group similar health care service providers by category labels.

The types of health care services that this study examined are listed in Table 1 and accompanied by keyword terms used to identify them in Yelp’s dataset (the results of the keyword searches are listed in Multimedia Appendix 1). This study focused on the types of health care services that were short term (ie, excluding those that required long-term physical residence such as rehabilitation facilities or nursing homes where accessibility and patient rating may interact differently), for human (ie, excluding those that care for animals such as veterinarians), for services that often require the provider to have minimum education or training equivalent to a Bachelor’s degree (ie, excluding health care services such as massage therapy), and the core business is to provide health care services (ie, excluding drugstores that often also function as convenience stores) (Table 1).

The health service providers were from six metropolitan areas: Pittsburgh, PA; Charlotte, NC; Urbana-Champaign, IL; Phoenix, AZ; Las Vegas, NV; and Madison, WI. It is worth noting that Yelp groups multiple cities or towns into a metropolitan area. Therefore, the health care service providers in our sample were not exclusively located within the six cities listed, but also from the surrounding cities and towns of those cities to comprise greater metropolitan areas. This study adopted Yelp’s metropolitan area grouping.

### Analysis

First, this study explored the pattern of patient visits to health care services by plotting Yelp’s check-in volume for each 1-hour window throughout the days of the week. Then, we examined if there were possible associations between patient rating and the variables extracted from Yelp, using ordinal logistic regression models with R package MASS [27]. This study considered significance level at *P* value ≤.05.

This study modeled the overall rating of the health care service provider as the dependent variable. The independent variables of primary interest were the number of opening hours during normal working hours and outside of normal working hours throughout the week. Although the rating may appear to be a continuous variable, this study assumed that it was an ordered categorical dependent variable because the numerical gap between consecutive categories may be inconsistent. For instance, the gap between 1 and 2 stars may be different than that between 3 and 4 stars. Therefore, we chose to use ordinal logistic regression as our main analytic method.

The following possible covariates found in the Yelp dataset were also modeled as independent variables: whether an appointment is required (true/false), the type of health care service (indicated by Yelp’s category label in Table 1), the region of the health care service provider (metro area listed on Yelp), and the number of reviews for each provider (review count provided by Yelp).

**Figure 1:**
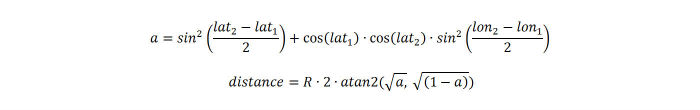
Equation for calculation the distance between a pair of providers using the geographical coordinates listed on Yelp, where (lat1, lon1) and (lat2, lon2) represent the latitude and longitude coordinates in radians of the two providers, and the radius of the Earth is 3961 miles.

Additionally, from the geographical coordinates listed on Yelp, we were able to derive the number of nearby competitors, the average rating of nearby competitors, and the variation in the ratings of nearby competitors, measured by standard deviation, as covariates. Competitors of each health care service provider were identified as providers of the same type as defined in Table 1 and within a 5-mile radius. The distance between a pair of providers is calculated using the geographical coordinates listed on Yelp in Figure 1 [28,29].

This study first evaluated a model of all the variables we were able to obtain from Yelp (model 1). Then we evaluated a limited model with only the continuous variables that showed a significant Pearson correlation to patient rating and categorical variables that showed a significant difference in mean rating among the groups using ANOVA, and without the mental health and speech therapy groups due to small sample size (model 2). Recognizing that different types of health care service may have different relationships between opening hours and rating, we evaluated stratified models for the types of health care service as well.

**Table 1:** Search keywords used to identify the health care service providers of interest by the category label in Yelp’s dataset.

Health care service type	Included if contained the following keyword(s)	Excluded if contained the following keyword(s)
Chiropractic and physical therapy	Chiropractor OR physical therapy	
Dental	Dentist	
Dermatology	Dermatologist	Optometrist, veterinarians, pets
Family practice	Family practice	Psychiatrist, chiropractor, beauty, physical therapy, specialty, dermatologists, weight loss, acupuncture, cannabis clinics, naturopathic, optometrists
Hospitals and clinics	Hospital	Physical therapy, rehab, retirement homes, veterinarians, dentist
Optometry	Optometrist	Dermatologist
Mental health	Psychiatrist OR psychologist	
Speech therapy	Speech	

### Opening Hours and Pattern of Visits

This study used the business hours (open and close times) listed on Yelp. The hours listed can be updated by either the owner of the business (if they are registered and verified by Yelp) or by any Yelp user who wishes to update the information.

Initially, this study examined the association between the total number of opening hours per week (Sunday to Saturday) and rating, but found that the linear regression model accounted for only 6.09% (*P*<.001) of the variation in the data. Additionally, the observation of the pattern of visits from the check-in data suggested that most of the visits to health care service providers took place between Sunday and Thursday and during regular working hours (9 am to 5 pm) on those days, and there was periodicity throughout the week. Therefore, the total number of opening hours per week may not be sufficiently granular, so this study considered the distribution of opening hours throughout the week to see if it was significantly correlated with patients’ ratings. Specifically, the total hours of operation on each day of the week were separated into the number of opening hours during normal working hours between 9 am to 5 pm (range 0-8 hours) and outside of normal working hours (range 0-16 hours) to be used as independent variables in the ordinal logistic regression.

## Results

### Sample Characteristics

The keyword search identified 3098 providers. This study then excluded providers that were cross-listed in more than one type of health care service after the keyword search defined in Table 1 due to ambiguity (n=22 or 11 unique records), were from outside the United States (n=46), did not specify whether an appointment was needed (n=237), without any opening hours listed (n=642), and had total opening hours per week less than or equal to zero (n=66). This left a total of 2085 eligible health service providers for this study. Filtering the dataset identified 31,356 check-in events associated with the health care service providers in the sample.

From the 2085 health care service providers in the sample, the mean rating was 4.18 stars (SD 0.91; median 4.5, range 1-5). The mean opening hours was 42.94 hours per week (SD 11.51; median 43, range 7.5-105). In all, 93.09% (1941/2085) of the health care service providers in the sample operated outside of normal working hours on at least one day per week. The mean opening hours outside of normal working hours was 7.11 hours per week (SD 5.30; median 6, range 0-49). Further descriptive statistics for each type of health care service is presented in Table 2.

**Table 2:** Summary of the proportion of the sample, mean rating, and review count for each type of health care service and appointment attributes from the sample of 2085 health care service providers.

Variables	Sample size, n (%)	Rating,^a^ mean (SD)	Review count, mean (SD)
Chiropractic/physical therapy	480 (23.02)	4.55 (0.69)	10.34 (10.89)
Dental	1014 (48.63)	4.31 (0.81)	11.68 (11.36)
Dermatology	88 (4.22)	3.43 (0.89)	17.54 (15.80)
Family practice	112 (5.37)	3.22 (0.92)	14.31 (15.01)
Hospitals/Clinics	20 (0.96)	3.33 (0.86)	10.75 (11.89)
Optometry	362 (17.36)	3.90 (0.98)	14.69 (14.17)
Mental health	7 (0.34)	3.00 (1.61)	6.43 (3.95)
Speech therapy	2 (0.10)	4.25 (1.06)	7.00 (4.24)
Appointment required	1580 (75.78)	4.17 (0.92)	11.68 (11.51)
Appointment not required	505 (24.22)	4.22 (0.87)	14.04 (14.48)

^a^ Mean rating range: 1-5 stars.

### Association Between Opening Hours and Patient Rating

**Figure 2:**
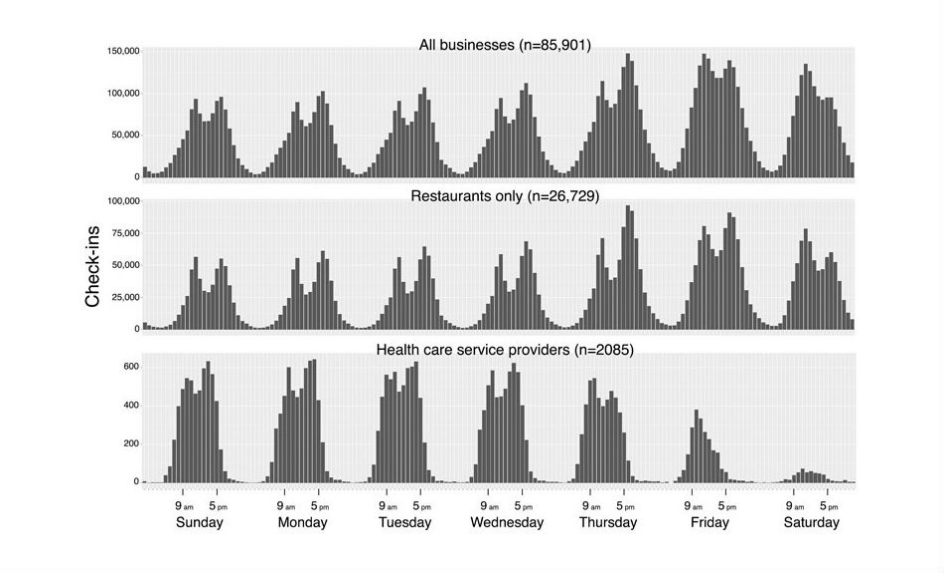
Number of check-ins for each 1-hour interval in the week for all businesses, restaurants, and health care service providers from Yelp’s limited dataset.

**Figure 3:**
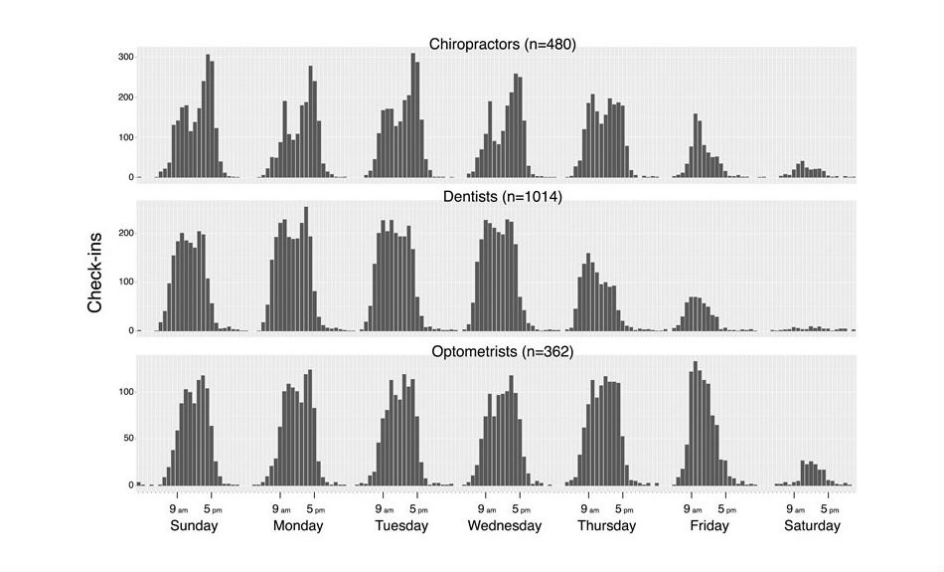
Number of check-ins for each 1-hour interval in the week for chiropractors/physical therapists, dentists, and optometrists from Yelp’s limited dataset.

The check-in data suggested that the volume of visits to health care service providers varied across the days of week (Figures 2 and 3). Contrary to other types of businesses on Yelp, most commonly restaurants, the majority of visits to health care service providers appeared to take place between Sunday and Thursday during normal working hours, and the volume decreased from Thursday to Saturday (Figure 2). Similar trends were also observed for the top three most common health care services in the sample (Figure 3). One slight deviation was optometry, where the volume of visits was relatively constant from Sunday to Friday, but the lowest volume was still on Saturday (Figure 3).

**Table 3:** Relative odds of achieving higher rating according to opening hours during different periods of the week and business characteristic variables available through Yelp’s limited dataset. Bold indicates *P* ≤.05.

Independent variables			Model 1 (full), OR (95% CI)	Model 2 (limited),^a^ OR (95% CI)	Model 3 (chiropractor/PT),^b^ OR (95% CI)	Model 4 (dentists),^c^ OR (95% CI)	Model 5 (optometrists),^d^ OR (95% CI)
**Number of opening hours during**							
	**9am-5pm**						
		Monday	**0.926 (0.880-0.973)**	**0.924 (0.878-0.971)**	1.124 (0.966-1.312)	**0.895 (0.837-0.955)**	**0.825 (0.728-0.930)**
		Tuesday	0.989 (0.927-1.056)	0.981 (0.919-1.046)	0.953 (0.849-1.068)	0.988 (0.898-1.085)	0.820 (0.639-1.034)
		Wednesday	1.007 (0.954-1.061)	1.003 (0.951-1.057)	0.913 (0.792-1.047)	1.015 (0.945-1.089)	0.906 (0.778-1.048)
		Thursday	0.997 (0.938-1.059)	0.997 (0.939-1.059)	0.985 (0.871-1.113)	0.975 (0.894-1.061)	1.068 (0.891-1.271)
		Friday	0.972 (0.936-1.008)	0.974 (0.939-1.010)	0.967 (0.873-1.069)	0.996 (0.949-1.045)	**0.838 (0.713-0.977)**
		Saturday	**0.897 (0.860-0.935)**	**0.907 (0.871-0.945)**	0.960 (0.868-1.063)	**0.866 (0.808-0.928)**	**0.876 (0.811-0.946)**
		Sunday	**0.904 (0.841-0.970)**	**0.906 (0.844-0.972)**	0.913 (0.794-1.050)	1.069 (0.910-1.256)	0.891 (0.783-1.014)
	**Outside 9am-5pm**						
		Monday	0.999 (0.851-1.172)	0.994 (0.847-1.166)	0.872 (0.564-1.343)	1.079 (0.884-1.317)	0.722 (0.435-1.214)
		Tuesday	1.047 (0.913-1.201)	1.062 (0.927-1.217)	1.249 (0.951-1.678)	0.967 (0.800-1.169)	**2.034 (1.321-3.167)**
		Wednesday	0.936 (0.802-1.092)	0.938 (0.804-1.094)	1.123 (0.762-1.655)	0.938 (0.768-1.144)	1.026 (0.671-1.581)
		Thursday	1.073 (0.922-1.250)	1.070 (0.919-1.246)	0.811 (0.559-1.164)	1.132 (0.931-1.378)	1.152 (0.750-1.779)
		Friday	**0.872 (0.760-0.998)**	**0.868 (0.758-0.993)**	0.990 (0.747-1.311)	**0.734 (0.601-0.895)**	**0.607 (0.385-0.945)**
		Saturday	0.882 (0.735-1.056)	0.876 (0.731-1.048)	0.989 (0.624-1.573)	1.001 (0.711-1.409)	0.818 (0.544-1.226)
		Sunday	**1.400 (1.036-1.924)**	1.338 (0.994-1.819)	1.290 (0.748-2.288)	0.695 (0.339-1.373)	0.948 (0.339-2.702)
**Other covariates**							
	Competitor count		1.001 (0.997-1.005)	1.003 (0.999-1.006)	1.004 (0.990-1.017)	1.002 (0.998-1.006)	1.016 (0.999-1.034)
	Competitors’ rating (mean)		1.052 (0.914-1.209)	1.102 (0.960-1.263)	1.113 (0.841-1.452)	1.038 (0.683-1.582)	0.808 (0.584-1.108)
	Competitors’ rating (SD)		**0.628 (0.458-0.862)**	**0.685 (0.501-0.935)**	**0.470 (0.220-0.998)**	0.748 (0.417-1.343)	0.622 (0.323-1.189)
	Appointment required(false)		Referent				
	Appointment required (true)		0.863 (0.706-1.055)				
	Review count		1.005 (0.999-1.012)				
**Health care service type**							
	Chiropractic/PT		Referent	Referent			
	Dental		**0.452 (0.343-0.594)**	**0.428 (0.326-0.560)**			
	Dermatology		**0.082 (0.050-0.132)**	**0.091 (0.057-0.146)**			
	Family practice		**0.059 (0.037-0.094)**	**0.066 (0.042-0.104)**			
	Hospitals/ clinics		**0.076 (0.032-0.182)**	**0.095 (0.040-0.226)**			
	Optometry		**0.355 (0.252-0.493)**	**0.364 (0.262-0.506)**			
	Mental health		**0.034 (0.007-0.189)**				
	Speech therapy		0.673 (0.034-24.028)				
**Metropolitan areas**							
	Phoenix, AZ		Referent				
	Urbana-Champaign, IL		1.772 (0.451-7.655)				
	Charlotte, NC		0.809 (0.563-1.168)				
	Las Vegas, NV		1.041 (0.855-1.266)				
	Pittsburgh, PA		1.325 (0.742-2.399)				
	Madison, WI		0.621 (0.381-1.014)				

^a^Model 2 excluded appointment requirement, metro area due to no significant difference in rating among the groups were found in ANOVA; review count due to no significant Pearson correlation was found with rating; mental health and speech therapy groups due to small sample sizes.

^b^Model 3 contains only chiropractors or physical therapists (n=480).

^c^Model 4 contains only dentists (n=1014).

^d^Model 5 contains only optometrists (n=362).

Table 3 tabulates the results of the five different logistic regression models: model 1 that contained all variables available, model 2 with limited number of covariates and types of health care service types, model 3 that stratified for chiropractors/physical therapists, model 4 that stratified for dentists, and model 5 that stratified for optometrists. Because there were 17 to 20 independent variables in each model, a sample of roughly 200 or larger was required to avoid overfitting. Therefore, we only investigated the three stratified models for groups (chiropractors/physical therapists, dentists, and optometrists) that had sufficient sample size.

In our first ordinal logistic regression model (model 1), patient rating appeared to have an inverse association with opening hours during normal working hours on Monday, Saturday, Sunday, and outside normal working hours on Friday; and a positive association with opening hours outside of normal working hours on Sunday (Table 3). There was a statistically significant association between patient rating and the type of health care service (Table 3). The results suggest that ratings are more likely to be higher for chiropractic/physical therapy than other types of health care services (Tables 2 and 3).

In model 2, the review count variable was removed due to a lack of significant correlation with patient rating. In addition, appointment requirement and metropolitan area were excluded due to a lack of significant difference in rating means among the groups, and mental health and speech therapy were also removed due to small sample sizes. Despite removing these variables, models 1 and 2 had similar patterns of association between opening hours and patient rating, except for opening hours outside of normal working hours on Sunday, which was only statistically significant in model 1 (Table 3). Furthermore, both models 1 and 2 suggested that the variation in competitors’ ratings measured in standard deviation was inversely associated with patient rating, whereas the number of nearby competitors and the mean rating of competitors were not (Table 3).

Separate models for the top three most common types of health care services in the sample in models 3 to 5 showed that the association between opening hours in different time periods and patient rating varied for different types of health care services (Table 3). In model 3, for chiropractic and physical therapy providers there appeared to be no association between opening hours in different time periods and patient rating, but the variation in competitors’ ratings still showed an inverse association with patient rating, as in model 2 (Table 3). In model 4, dental providers also showed an inverse association between patient rating and opening hours during normal working hours on Monday, Saturday, and outside normal working hours on Friday (Table 3). In model 5, optometrists showed an inverse association between patient rating and opening hours during normal working hours on Monday, Friday, Saturday, and outside normal working hours on Friday; and positive association between patient rating and opening hours outside of business hours on Tuesday (Table 3).

## Discussion

### Association Between Opening Hours and Patient Rating

The check-in data and our ordinal logistic regression models consistently suggest that increasing the number of opening hours alone does not immediately lead to an impact on patient rating, and the impact may be specific to only certain time periods of the week (Figures 2 and 3,Table 3). The results from our study generally align with most previous findings. A previous study in the United Kingdom found that patient satisfaction rating in a survey was related to increasing opening hours, but was not linked to a specific time period [1]. Two other studies in the United Kingdom found that the ability to take time off from work to access health care may influence satisfaction ratings on a survey [2,30]. A survey conducted in Quebec, Canada, also found that increasing the total clinic opening hours per week may not immediately lead to an increase in patient rating, and the distribution of hours throughout the week may be more important [7]. Other factors such as the number of physicians, 24/7 telephone access, evening walk-in, and care are also important [7].

The difference in whether the association between patient rating and opening hours is linked to specific periods of the week could be due to the differences in the sample populations. It is possible that the results of our study are more aligned with the sample population in Quebec, Canada, than the United Kingdom sample because our sample population is also from North America.

It appears that there may be unmet demand for Sunday, when there is a positive association between patient rating and extended hours on Sunday (Figure 3 and Table 3, model 1). If health care providers only offer opening hours during normal working hours on Sunday, some patients may feel restricted in options in terms of the hours, which may cause ratings to be lower in that period and lead to inverse association for Sunday during normal working hours (Table 3, models 1 and 2). It is also plausible that those who seek to access health care on Sunday but cannot find any open business may have to defer to working days, and the ability to take time off from work has been shown to be linked to patient rating [2,30].

On the other hand, models 1 and 2 suggest an inverse association between patient ratings and Saturday normal working hours and Friday outside normal working hours. No previous study has explored this topic in detail, but one plausible explanation may be that Friday night and Saturday are often perceived as time for family, leisure activities, or relaxation in most North American cultures. This was similarly observed from the check-in data that showed an increase in visits to restaurants and all businesses on Friday and Saturday (Figure 3).

### Appointment

The need for an appointment did not show an association with patient rating in model 1, despite previous studies suggesting that it may be important to patient satisfaction rating, demand, and accessibility [1,7,30,31]. A possible explanation is that patients who require service immediately and are unable to make an appointment with a provider would not visit that provider. Consequently, they would not write a review for the provider whom they failed to visit; hence, the effect of the need of advanced appointment being a barrier to access may not have been captured. Given this speculation, Yelp data may be limited in its ability to explore this topic.

### Type of Health Care Service

All our models suggest that the association between patient rating and opening hours varies across different types of health care services (Table 3). This was expected because there is a lot of variation in health care services. The relationship between patient rating and the type of health care service is an interesting area for future research that could help health care professionals improve their practices.

This study categorized different types of health care service into their subgroups to reduce possible confounding related to the types of health care services. The categorization was based on initial observation of the available dataset, and we tried to avoid overcategorization that could result in too few cases in each subgroup to produce any significant results (Table 1).

On the other hand, there may be challenges in categorizing health care services for future research based on Yelp-assigned labels. A nonspecific label such as “doctors” is ambiguous in identifying a specific specialist or to infer the type of health care service. Since Yelp allows any business to have multiple labels, there may be an incentive for overlabeling to ensure that a business appears more frequently in search results. As a consequence, future research should be aware of the ambiguity and uncertainty in categorizing health care services associated with using Yelp data. An additional verification step to ensure the accuracy of the category label is recommended.

### Competition

The inverse association between patient rating and the variation in competitors’ ratings suggests that competition may play a role in patient rating. We chose a radius of 5 miles for nearby competitors in this study, but this may also depend on various factors such as type of service, population density, health insurance’s network of providers, etc. Therefore, the results may vary as the radius is changed.

### Strengths and Limitations

The main contribution of this study is two-fold. First, surveying data was substituted by Yelp data and our results support previous findings that the distribution of opening hours may be more important to patient rating than simply total opening hours per week [1,7,30]. Second, this study provided an in-depth investigation of the distribution of opening hours and patient ratings, which to our knowledge had not been studied at this level of granularity.

However, this study had several limitations in sample size, geographical grouping, cross-sectional design, and the variables available through Yelp. Our sample population is likely to be younger people from only large metropolitan areas [22,32]. Therefore, they may have a very different pattern of health care service utilization than the general United States population. In addition, in the academic dataset, Yelp provides data only for businesses with three or more reviews older than 14 days at the time of data extraction. As a result, data quality may have been enhanced in terms of patient ratings, but this certainly is a limitation compared to the full dataset [22]. This study adopted Yelp’s regional grouping for the metro areas; we are not certain of their rationale, but suspect that it was based on proximity. Because this dataset was only a snapshot of the health care services, the results from this study can only infer cross-sectional associations, rather than causation, between patient rating and the independent variables.

This study was able to account for some possible confounding factors of patient satisfaction because the Yelp dataset is limited in the number of variables, which is often the case with many datasets and surveys. Compared to a traditional survey method, this study does not have access to the commonly collected demographic variables such as gender, age, and socioeconomic status. Therefore, Yelp data are limited in the ability to control for such covariates.

Furthermore, patient satisfaction and opinion rating remain complex, multifaceted concepts [13,33]. One commonly cited definition of a patient satisfaction rating by Ware and colleagues [34] states: satisfaction rating is “an attempt to capture a personal evaluation of care” reflecting “the personal preferences” of the patient, “the patient’s expectations,” and “the reality of care received.” Yelp’s guidelines asked users for their “firsthand consumer experience” [35]. Therefore, Yelp ratings appear to be a close proxy for patient satisfaction as defined in the literature. Our study detected some possible patient preferences for opening hours, but was limited in providing insights into patients’ expectations and reality of care.

### Future Work

There are opportunities for future research and to overcome the limitations outlined. Future research should expand the sample size beyond the current available dataset. Other methods of regional grouping may yield additional insights and is worth exploring in future research. Since Yelp’s data contain rich geographical data, there is also an opportunity to link to other datasets, such as census data, that may inform future research design. A longitudinal study could be conducted to shed more light on causality using multiple snapshots of the Yelp dataset over time. Although the variables used in this study may not be able to provide insights about differences among health services, the wealth of information in Yelp’s free-text comments could be useful for future research. The free-text reviews submitted by Yelp users can be used to extract more information that could be associated with patient rating and help to explore more dimensions of patient satisfaction.

### Conclusions

An association between opening hours of health care service providers and patient rating was observed from Yelp’s limited dataset. In the context of our sample, the observed association appears to vary and is specific to only certain time periods of the week. Therefore, increasing opening hours alone as an attempt to influence patient rating or satisfaction, without considering patient demand or preference, may not be effective. Other factors, such as the type of health care service and ratings of nearby health care providers, may also be related to patient rating and further research is needed.

Yelp data demonstrate the use of crowdsourced open Internet data can complement and potentially replace traditional surveying methods to some extent. The knowledge generated from Yelp can complement and enhance the evidence base for decision making and evaluation of public health interventions. This study hopes to catalyze further exploration of publicly available online data for health research.

## Appendix

Multimedia Appendix 1 Resulting categories found from Yelp’s dataset using the search terms in Table 1 and after applying the inclusion and exclusion criteria.

## Abbreviations

HCAHPS: Hospital Consumer Assessment of Healthcare Providers and Systems
NHS: National Health Service
