# Supplementary material for: Online Reviews as Health Data: Examining the Association Between Availability of Health Care Services and Patient Star Ratings Exemplified by the Yelp Academic Dataset
Source: JMIR Public Health Surveill. 2017 Jul 12;3(3):e43. doi: 10.2196/publichealth.7001 (PMC5529738; doi:10.2196/publichealth.7001)
Supplement: Multimedia Appendix 1 [file publichealth_v3i3e43_app1.pdf]

Appendix

Appendix 1. Resulting categories found from Yelp's dataset using the search terms in Table 1 and after applying the inclusion and exclusion criteria.

Type of Healthcare Service	Search Results
Chiropractic/PT	[1] "Active Life, Gyms, Health & Medical, Physical Therapy, Fitness & Instruction"
	[2] "Active Life, Health & Medical, Yoga, Physical Therapy, Fitness & Instruction"
	[3] "Active Life, Nutritionists, Health & Medical, Chiropractors, Fitness & Instruction"
	[4] "Active Life, Pilates, Health & Medical, Physical Therapy, Fitness & Instruction"
	[5] "Acupuncture, Doctors, Health & Medical, Chiropractors, Naturopathic/Holistic"
	[6] "Acupuncture, Doctors, Health & Medical, Naturopathic/Holistic, Chiropractors"
	[7] "Acupuncture, Doctors, Sports Medicine, Health & Medical, Chiropractors"
	[8] "Acupuncture, Health & Medical, Chiropractors"
	[9] "Acupuncture, Health & Medical, Massage, Chiropractors, Beauty & Spas"
	[10] "Acupuncture, Health & Medical, Physical Therapy, Chiropractors"
	[11] "Acupuncture, Massage Therapy, Health & Medical, Chiropractors"
	[12] "Acupuncture, Massage Therapy, Health & Medical, Physical Therapy"
	[13] "Acupuncture, Medical Centers, Health & Medical, Chiropractors"
	[14] "Beauty & Spas, Health & Medical, Chiropractors, Medical Spas"
	[15] "Beauty & Spas, Health & Medical, Physical Therapy, Chiropractors, Medical Spas"
	[16] "Chiropractors, Beauty & Spas, Laser Hair Removal, Massage Therapy, Health & Medical, Hair Removal"
	[17] "Chiropractors, Beauty & Spas, Medical Spas, Skin Care, Health & Medical, Massage"
	[18] "Diagnostic Services, Health & Medical, Chiropractors"
	[19] "Doctors, Chiropractors, Naturopathic/Holistic, Beauty & Spas, Medical Spas, Health & Medical"
	[20] "Doctors, Health & Medical, Chiropractors"
	[21] "Doctors, Health & Medical, Chiropractors, Pain Management"

[22] "Doctors, Health & Medical, Family Practice, Chiropractors, Physical Therapy"

[23] "Doctors, Health & Medical, Naturopathic/Holistic, Chiropractors"

[24] "Doctors, Health & Medical, Naturopathic/Holistic, Chiropractors, Physical Therapy"

[25] "Doctors, Health & Medical, Physical Therapy, Urgent Care"

[26] "Doctors, Health & Medical, Urgent Care, Chiropractors"

[27] "Doctors, Medical Centers, Health & Medical, Physical Therapy, Family Practice"

[28] "Doctors, Medical Centers, Health & Medical, Physical Therapy, Orthopedists"

[29] "Doctors, Nutritionists, Chiropractors, Naturopathic/Holistic, Health & Medical, Acupuncture"

[30] "Doctors, Nutritionists, Health & Medical, Naturopathic/Holistic, Chiropractors"

[31] "Doctors, Nutritionists, Health & Medical, Physical Therapy, Family Practice"

[32] "Doctors, Orthopedists, Health & Medical, Chiropractors"

[33] "Doctors, Orthopedists, Health & Medical, Physical Therapy"

[34] "Doctors, Orthopedists, Health & Medical, Physical Therapy, Podiatrists"

[35] "Doctors, Physical Therapy, Beauty & Spas, Health & Medical, Sports Medicine, Massage"

[36] "Health & Medical, Chiropractors"

[37] "Health & Medical, Lactation Services, Chiropractors"

[38] "Health & Medical, Massage, Chiropractors, Beauty & Spas"

[39] "Health & Medical, Physical Therapy"

[40] "Health & Medical, Physical Therapy, Chiropractors"

[41] "Health & Medical, Physical Therapy, Massage, Beauty & Spas"

[42] "Massage Therapy, Acupuncture, Health & Medical, Chiropractors"

[43] "Massage Therapy, Beauty & Spas, Health & Medical, Chiropractors, Medical Spas"

[44] "Massage Therapy, Beauty & Spas, Health & Medical, Physical Therapy, Medical Spas"

[45] "Massage Therapy, Doctors, Health & Medical, Chiropractors, Pain Management"

[46] "Massage Therapy, Doctors, Health & Medical, Naturopathic/Holistic, Chiropractors"

[47] "Massage Therapy, Health & Medical, Chiropractors"

[48] "Massage Therapy, Health & Medical, Massage, Chiropractors, Beauty & Spas"

[49] "Massage Therapy, Health & Medical, Physical Therapy"

[50] "Massage Therapy, Health & Medical, Physical Therapy, Chiropractors"

[51] "Massage Therapy, Nutritionists, Health & Medical, Chiropractors"

[52] "Massage Therapy, Rehabilitation Center, Health & Medical, Physical Therapy"

[53] "Massage Therapy, Shopping, Health & Medical, Chiropractors, Vitamins & Supplements"

[54] "Medical Centers, Health & Medical, Chiropractors"

[55] "Medical Centers, Health & Medical, Physical Therapy"

[56] "Medical Centers, Health & Medical, Physical Therapy, Chiropractors"

[57] "Neurologist, Doctors, Health & Medical, Chiropractors"

[58] "Nutritionists, Chiropractors, Health & Medical, Trainers, Fitness & Instruction, Active Life"

[59] "Nutritionists, Health & Medical, Chiropractors"

[60] "Nutritionists, Health & Medical, Medical Centers, Chiropractors"

[61] "Nutritionists, Health & Medical, Physical Therapy, Chiropractors"

[62] "Nutritionists, Physical Therapy, Health & Medical, Trainers, Fitness & Instruction, Active Life"

[63] "Occupational Therapy, Health & Medical, Physical Therapy"

[64] "Physical Therapy, Health & Medical, Massage, Chiropractors, Beauty & Spas"

[65] "Pilates, Physical Therapy, Chiropractors, Health & Medical, Fitness & Instruction, Active Life"

[66] "Pilates, Physical Therapy, Health & Medical, Trainers, Fitness & Instruction, Active Life"

[67] "Rehabilitation Center, Doctors, Health & Medical, Neurologist, Chiropractors"

[68] "Rehabilitation Center, Health & Medical, Chiropractors"

[69] "Rehabilitation Center, Health & Medical, Physical Therapy"

	[70] "Rehabilitation Center, Health & Medical, Physical Therapy, Chiropractors"<br><br>[71] "Sports Medicine, Doctors, Health & Medical, Chiropractors"<br><br>[72] "Sports Medicine, Doctors, Health & Medical, Chiropractors, Rehabilitation Center"<br><br>[73] "Sports Medicine, Doctors, Health & Medical, Naturopathic/Holistic, Chiropractors"<br><br>[74] "Sports Medicine, Doctors, Health & Medical, Physical Therapy"<br><br>[75] "Sports Medicine, Doctors, Health & Medical, Physical Therapy, Chiropractors"<br><br>[76] "Sports Medicine, Doctors, Health & Medical, Physical Therapy, Pain Management"<br><br>[77] "Sports Medicine, Doctors, Massage Therapy, Health & Medical, Chiropractors"<br><br>[78] "Sports Medicine, Doctors, Massage Therapy, Health & Medical, Physical Therapy"<br><br>[79] "Sports Medicine, Doctors, Orthopedists, Health & Medical, Physical Therapy"<br><br>[80] "Weight Loss Centers, Doctors, Health & Medical, Naturopathic/Holistic, Chiropractors"<br><br>[81] "Weight Loss Centers, Health & Medical, Chiropractors"<br><br>[82] "Weight Loss Centers, Health & Medical, Physical Therapy"<br><br>[83] "Weight Loss Centers, Health & Medical, Physical Therapy, Chiropractors"<br><br>[84] "Weight Loss Centers, Massage Therapy, Health & Medical, Chiropractors"<br><br>[85] "Weight Loss Centers, Nutritionists, Health & Medical, Chiropractors"<br><br>[86] "Weight Loss Centers, Orthotics, Health & Medical, Chiropractors"<br><br>[87] "Yoga, Chiropractors, Beauty & Spas, Health & Medical, Fitness & Instruction, Active Life, Massage"<br><br>[88] "Yoga, Physical Therapy, Health & Medical, Trainers, Fitness & Instruction, Active Life"
Dental	[1] "Dentists, Beauty & Spas, Shopping, Health & Medical, Cosmetics & Beauty Supply, Cosmetic Dentists"<br><br>[2] "Dentists, Doctors, Health & Medical, Cosmetic Surgeons, General Dentistry"<br><br>[3] "Dentists, Doctors, Health & Medical, Cosmetic Surgeons, Oral Surgeons"<br><br>[4] "Dentists, Financial Services, Health & Medical, Orthodontists, General Dentistry, Cosmetic Dentists, Insurance"

[5] "Dentists, Health & Medical, General Dentistry, Urgent Care, Cosmetic Dentists"

[6] "Dentists, Teeth Whitening, Beauty & Spas, Health & Medical, General Dentistry, Cosmetic Dentists"

[7] "Dentists, Teeth Whitening, Beauty & Spas, Periodontists, Health & Medical, General Dentistry"

[8] "Doctors, Dentists, Cosmetic Surgeons, Health & Medical, General Dentistry, Cosmetic Dentists"

[9] "Doctors, Dentists, Cosmetic Surgeons, Oral Surgeons, Health & Medical, General Dentistry"

[10] "Endodontists, Health & Medical, Dentists"

[11] "Endodontists, Health & Medical, Dentists, General Dentistry"

[12] "Endodontists, Health & Medical, Dentists, General Dentistry, Cosmetic Dentists"

[13] "Endodontists, Health & Medical, Dentists, Oral Surgeons"

[14] "Endodontists, Health & Medical, Dentists, Orthodontists, Cosmetic Dentists"

[15] "Endodontists, Health & Medical, Dentists, Orthodontists, General Dentistry"

[16] "Endodontists, Health & Medical, Periodontists, Dentists, Cosmetic Dentists"

[17] "Endodontists, Health & Medical, Periodontists, Dentists, General Dentistry"

[18] "Endodontists, Health & Medical, Periodontists, Dentists, Orthodontists"

[19] "Health & Medical, Dentists"

[20] "Health & Medical, Dentists, Cosmetic Dentists"

[21] "Health & Medical, Dentists, General Dentistry"

[22] "Health & Medical, Dentists, General Dentistry, Cosmetic Dentists"

[23] "Health & Medical, Dentists, Orthodontists"

[24] "Health & Medical, Dentists, Orthodontists, Cosmetic Dentists"

[25] "Health & Medical, Dentists, Orthodontists, General Dentistry"

[26] "Health & Medical, Dentists, Orthodontists, General Dentistry, Cosmetic Dentists"

[27] "Health & Medical, Dentists, Sleep Specialists, General Dentistry, Cosmetic Dentists"

[28] "Health & Medical, Dentists, Urgent Care, General Dentistry"

[29] "Health & Medical, Periodontists, Dentists"

[30] "Health & Medical, Periodontists, Dentists, Cosmetic Dentists"

[31] "Health & Medical, Periodontists, Dentists, General Dentistry"

[32] "Health & Medical, Periodontists, Dentists, General Dentistry, Cosmetic Dentists"

[33] "Health & Medical, Periodontists, Dentists, Orthodontists, Cosmetic Dentists"

[34] "Health & Medical, Periodontists, Dentists, Orthodontists, General Dentistry"

[35] "Health & Medical, Prosthodontists, Dentists, General Dentistry, Cosmetic Dentists"

[36] "Oral Surgeons, Health & Medical, Dentists"

[37] "Oral Surgeons, Health & Medical, Dentists, Cosmetic Dentists"

[38] "Oral Surgeons, Health & Medical, Dentists, Cosmetic Surgeons, Doctors"

[39] "Oral Surgeons, Health & Medical, Dentists, General Dentistry"

[40] "Oral Surgeons, Health & Medical, Dentists, General Dentistry, Cosmetic Dentists"

[41] "Oral Surgeons, Health & Medical, Dentists, Orthodontists"

[42] "Oral Surgeons, Health & Medical, Dentists, Orthodontists, Cosmetic Dentists"

[43] "Oral Surgeons, Health & Medical, Dentists, Orthodontists, General Dentistry"

[44] "Oral Surgeons, Health & Medical, Dentists, Orthodontists, Pediatric Dentists"

[45] "Oral Surgeons, Health & Medical, Dentists, Pediatric Dentists, General Dentistry"

[46] "Oral Surgeons, Health & Medical, Endodontists, Dentists"

[47] "Oral Surgeons, Health & Medical, Endodontists, Dentists, General Dentistry"

[48] "Oral Surgeons, Health & Medical, Pain Management, Dentists, Doctors"

[49] "Oral Surgeons, Health & Medical, Periodontists, Dentists, Cosmetic Dentists"

[50] "Oral Surgeons, Health & Medical, Periodontists, Dentists, General Dentistry"

[51] "Oral Surgeons, Health & Medical, Prosthodontists, Dentists"

[52] "Pediatric Dentists, Health & Medical, Dental Hygienists, Dentists, Cosmetic Dentists"

[53] "Pediatric Dentists, Health & Medical, Dentists"

[54] "Pediatric Dentists, Health & Medical, Dentists, Doctors"

[55] "Pediatric Dentists, Health & Medical, Dentists, General Dentistry"

	[56] "Pediatric Dentists, Health & Medical, Dentists, General Dentistry, Cosmetic Dentists"<br><br>[57] "Pediatric Dentists, Health & Medical, Dentists, Oral Surgeons, Cosmetic Dentists"<br><br>[58] "Pediatric Dentists, Health & Medical, Dentists, Oral Surgeons, General Dentistry"<br><br>[59] "Pediatric Dentists, Health & Medical, Dentists, Orthodontists"<br><br>[60] "Pediatric Dentists, Health & Medical, Dentists, Orthodontists, Cosmetic Dentists"<br><br>[61] "Pediatric Dentists, Health & Medical, Dentists, Orthodontists, General Dentistry"<br><br>[62] "Pediatric Dentists, Health & Medical, Endodontists, Dentists, General Dentistry"<br><br>[63] "Pediatric Dentists, Health & Medical, Periodontists, Dentists, Cosmetic Dentists"<br><br>[64] "Pediatric Dentists, Health & Medical, Periodontists, Dentists, General Dentistry"<br><br>[65] "Pediatric Dentists, Health & Medical, Periodontists, Dentists, Orthodontists"
Dermatology	[1] "Dermatologists, Doctors, Beauty & Spas, Health & Medical, Medical Spas"<br><br>[2] "Dermatologists, Doctors, Hair Removal, Health & Medical, Beauty & Spas"<br><br>[3] "Dermatologists, Doctors, Health & Medical"<br><br>[4] "Dermatologists, Doctors, Health & Medical, Cosmetic Surgeons"<br><br>[5] "Dermatologists, Doctors, Health & Medical, Cosmetic Surgeons, Tattoo Removal"<br><br>[6] "Dermatologists, Doctors, Health & Medical, Internal Medicine, Pediatricians"<br><br>[7] "Dermatologists, Doctors, Health & Medical, Skin Care, Beauty & Spas"<br><br>[8] "Dermatologists, Doctors, Health & Medical, Surgeons"<br><br>[9] "Dermatologists, Doctors, Health & Medical, Tattoo Removal"<br><br>[10] "Dermatologists, Doctors, Health & Medical, Urgent Care, Cardiologists"<br><br>[11] "Dermatologists, Doctors, Health & Medical, Weight Loss Centers, Family Practice"<br><br>[12] "Dermatologists, Doctors, Medical Centers, Health & Medical, Weight Loss Centers"<br><br>[13] "Doctors, Beauty & Spas, Hair Removal, Skin Care, Health & Medical, Laser Hair Removal, Dermatologists"

	[14] "Doctors, Beauty & Spas, Laser Hair Removal, Skin Care, Health & Medical, Hair Removal, Dermatologists"<br><br>[15] "Doctors, Beauty & Spas, Medical Spas, Skin Care, Health & Medical, Dermatologists"<br><br>[16] "Doctors, Beauty & Spas, Skin Care, Health & Medical, Dermatologists, Tattoo Removal"<br><br>[17] "Doctors, Cosmetic Surgeons, Beauty & Spas, Laser Hair Removal, Health & Medical, Hair Removal, Dermatologists"<br><br>[18] "Doctors, Cosmetic Surgeons, Beauty & Spas, Medical Spas, Health & Medical, Dermatologists"<br><br>[19] "Doctors, Cosmetic Surgeons, Beauty & Spas, Medical Spas, Health & Medical, Pediatricians, Dermatologists"<br><br>[20] "Doctors, Cosmetic Surgeons, Beauty & Spas, Skin Care, Health & Medical, Dermatologists"<br><br>[21] "Doctors, Medical Centers, Beauty & Spas, Skin Care, Health & Medical, Dermatologists"<br><br>[22] "Doctors, Shopping, Beauty & Spas, Health & Medical, Cosmetics & Beauty Supply, Hair Removal, Dermatologists"<br><br>[23] "Doctors, Shopping, Beauty & Spas, Skin Care, Health & Medical, Cosmetics & Beauty Supply, Dermatologists"
Family Practice	[1] "Doctors, Family Practice, Health & Medical, Pediatricians, Urgent Care"<br><br>[2] "Doctors, Health & Medical, Family Practice"<br><br>[3] "Doctors, Health & Medical, Family Practice, Internal Medicine"<br><br>[4] "Doctors, Health & Medical, Family Practice, Urgent Care"<br><br>[5] "Doctors, Health & Medical, Home Health Care, Family Practice"<br><br>[6] "Doctors, Health & Medical, Internal Medicine, Family Practice"<br><br>[7] "Doctors, Health & Medical, Internal Medicine, Family Practice, Gerontologists"<br><br>[8] "Doctors, Health & Medical, Internal Medicine, Pediatricians, Family Practice"<br><br>[9] "Doctors, Health & Medical, Internal Medicine, Urgent Care, Family Practice"<br><br>[10] "Doctors, Health & Medical, Pediatricians, Family Practice"<br><br>[11] "Doctors, Health & Medical, Pediatricians, Family Practice, Internal Medicine"<br><br>[12] "Doctors, Health & Medical, Pediatricians, Family Practice, Urgent Care"<br><br>[13] "Doctors, Health & Medical, Urgent Care, Family Practice"

	[14] "Doctors, Medical Centers, Health & Medical, Family Practice"<br><br>[15] "Doctors, Medical Centers, Health & Medical, Internal Medicine, Family Practice"<br><br>[16] "Doctors, Medical Centers, Health & Medical, Pediatricians, Family Practice"<br><br>[17] "Doctors, Occupational Therapy, Health & Medical, Urgent Care, Family Practice"<br><br>[18] "Sports Medicine, Doctors, Health & Medical, Obstetricians & Gynecologists, Family Practice"
Hospitals/Clinics	[1] "Doctors, Hospitals, Health & Medical"<br><br>[2] "Doctors, Hospitals, Health & Medical, Internal Medicine"<br><br>[3] "Doctors, Hospitals, Health & Medical, Obstetricians & Gynecologists"<br><br>[4] "Doctors, Hospitals, Health & Medical, Urgent Care"<br><br>[5] "Health & Medical, Hospitals"<br><br>[6] "Health & Medical, Hospitals, Urgent Care"<br><br>[7] "Hospitals, Medical Centers, Health & Medical"
Optometry	[1] "Doctors, Laser Eye Surgery/Lasik, Ophthalmologists, Health & Medical, Optometrists"<br><br>[2] "Doctors, Ophthalmologists, Health & Medical, Optometrists"<br><br>[3] "Doctors, Ophthalmologists, Health & Medical, Optometrists, Laser Eye Surgery/Lasik"<br><br>[4] "Doctors, Shopping, Optometrists, Ophthalmologists, Health & Medical, Eyewear & Opticians"<br><br>[5] "Fashion, Shopping, Optometrists, Accessories, Health & Medical, Eyewear & Opticians"<br><br>[6] "Medical Centers, Shopping, Optometrists, Sporting Goods, Health & Medical, Outdoor Gear"<br><br>[7] "Optometrists, Health & Medical"<br><br>[8] "Optometrists, Health & Medical, Laser Eye Surgery/Lasik"<br><br>[9] "Optometrists, Health & Medical, Shopping, Eyewear & Opticians"<br><br>[10] "Optometrists, Health & Medical, Shopping, Eyewear & Opticians, Laser Eye Surgery/Lasik"<br><br>[11] "Optometrists, Laser Eye Surgery/Lasik, Ophthalmologists, Health & Medical, Doctors"<br><br>[12] "Optometrists, Medical Centers, Health & Medical"

	[13] "Optometrists, Medical Centers, Health & Medical, Shopping, Eyewear & Opticians"<br><br>[14] "Optometrists, Ophthalmologists, Health & Medical, Doctors"<br><br>[15] "Optometrists, Ophthalmologists, Health & Medical, Doctors, Laser Eye Surgery/Lasik"<br><br>[16] "Shopping, Optometrists, Health & Medical, Eyewear & Opticians"<br><br>[17] "Shopping, Optometrists, Health & Medical, Eyewear & Opticians, Doctors"<br><br>[18] "Shopping, Optometrists, Health & Medical, Eyewear & Opticians, Laser Eye Surgery/Lasik"<br><br>[19] "Shopping, Optometrists, Medical Centers, Health & Medical, Eyewear & Opticians"
Mental Health	[1] "Doctors, Addiction Medicine, Counseling & Mental Health, Health & Medical, Psychiatrists"<br><br>[2] "Doctors, Counseling & Mental Health, Health & Medical, Psychiatrists"<br><br>[3] "Doctors, Health & Medical, Psychiatrists"<br><br>[4] "Health & Medical, Counseling & Mental Health, Psychologists"<br><br>[5] "Sports Psychologists, Counseling & Mental Health, Health & Medical"
Speech Therapy	[1] "Speech Therapists, Health & Medical"<br><br>[2] "Yoga, Beauty & Spas, Speech Therapists, Health & Medical, Fitness & Instruction, Active Life, Massage"
